# Would it be legally justified to impose vaccination in Israel? Examining the issue in light of the 2013 detection of polio in Israeli sewage

**DOI:** 10.1186/s13584-017-0182-z

**Published:** 2017-10-30

**Authors:** Shelly Kamin-Friedman

**Affiliations:** Department of Health Systems Management, Faculty of Health Sciences, Ben-Gurion University of the Negev, Beer Sheva, Israel

**Keywords:** Polio, Public health law, Autonomy

## Abstract

**Background:**

The detection of wild poliovirus in Israeli sewage in May 2013 led the health authorities to decide that children who had been vaccinated with IPV would also be vaccinated with OPV. The decision sought to protect vulnerable Israeli individuals who were either not vaccinated with IPV or who suffered from an immune deficiency, to preserve Israel’s status as a polio-free country, to prevent the virus’ “exportation” into vulnerable polio-free countries, and to participate in the global efforts toward the eradication of polio. After a massive public persuasion campaign, 79% of the children born after 2004 were vaccinated as well as 69% of the children residing in central Israel. A 2014 State Comptroller Report stated that the Ministry of Health should draw conclusions from the low compliance rates in certain Israeli regions.

**Goals:**

The article seeks to examine the legal legitimacy of mandatory vaccination in the service of eradicating a contagious disease (as opposed to preventing a pandemic outbreak), which was one of the objectives in the 2013 Polio case. It more specifically relates to current Israeli law as well as to a hypothetical new public health law which would authorize health officials to oblige vaccination and enforce this through the use of criminal sanctions.

**Method:**

Qualitative content analysis through the interpretation of court judgements, laws, legislative protocols, health ministry guidelines and documented discussions of the Advisory Committee on Infectious Diseases and Immunization.

**Main findings and conclusion:**

A mandatory vaccination backed by criminal sanctions in the service of the eradication of contagious diseases would probably be perceived as infringing on the constitutional right to autonomy to a greater extent than necessary according to Israeli law and case law precedents. There may be some added value inherent in a new public health law which would authorize health officials to oblige vaccination where nonrestrictive measures have been ineffective. However, the law should also specify a variety of sanctions to accompany the enforcement of mandatory vaccinations which would be formulated from least to most restrictive according to the “intervention ladder” concept. The law should also describe the circumstances which would justify the implementation of each and every sanction as well as the procedural safeguards designed for established decisions and fairness toward the individual(s) whose rights are infringed by the application of these sanctions.

## Background: Israeli polio vaccination policy and the 2013 detection of polio in Israeli sewage

Polio is a severe disease which may cause paralysis. Two types of vaccines have been used against it since the 1950s and 1960s: IPV – inactivated polio vaccine, which induces humoral immunity but does not prevent intestinal infection, and OPV – an attenuated oral polio vaccine which induces a local and mucosal immune response in the intestinal mucous membrane and is later excreted. It thus not only protects the individual but can also be spread to others in close contact with the vaccinated individual and induce the “incidental” immunization of people who have not been directly vaccinated. A recipient of an OPV or an unimmunized close contact may rarely develop paralytic polio as a result of the vaccine. However, giving an OPV to someone already immunized with an IPV is very safe [1].

Israel started vaccinating children against polio in 1957. The immunization schedule changed according to developments in both OPV and IPV vaccines and according to epidemiological considerations. After the 1988 outbreak of the disease, Israeli children were routinely vaccinated with a combination of OPV and IPV. The vaccine did indeed significantly reduce polio morbidity. A total of 8 cases of VAPP – vaccine associated paralytic poliomyelitis have been reported between the beginning of monitoring in 1972 and 1985, when the last recorded case of VAPP was diagnosed. 6 of these were diagnosed in vaccine recipients and 2 in people who were in contact with vaccines [2].

Since there have been no cases of polio in Israel for years, and since the WHO recognized Israel as a polio-free country, Israeli children have, in accordance with WHO guidelines [3], been vaccinated with IPV alone since 2005.

In May 2013, and due to the consistent detection of wild poliovirus in Israeli sewage in several sample and growing concentrations, Israeli health authorities made efforts to reach unvaccinated children and vaccinate them with IPV. However, these efforts did not stop the environmental spread of the virus. In June 2013, a WHO delegation to Israel, the CDC and the Israeli polio committee advised that children who had been vaccinated with IPV since 2005 should also be vaccinated with OPV. On August 5, 2013 the parents of children in southern Israel who were born after 2004 were asked to vaccinate them with OPV. The recommendation was later extended to cover all Israeli parents of children born after 2004 since the wild poliovirus had been detected in other areas too [4].

The objectives of adding OPV to Israeli children already vaccinated with IPV were the protection of vulnerable Israeli individuals who were not vaccinated with IPV or who suffered from immune deficiency, the preservation of Israel’s status as a polio-free country, the prevention of the virus’ “exportation” to vulnerable polio-free countries, and participation in the global efforts toward the eradication of polio. It should, however, be noted that the IPV had been routinely administrated to more than 98% of Israeli children by the time the wild poliovirus was detected in Israeli sewage. As IPV decreases both the risk of infection and infectiousness [5], its high coverage prevented a polio outbreak in Israel [6–8].

In order to promote compliance with OPV, and proceeding from an understanding that the main policy communication challenge would lie in persuading parents to vaccinate their children for the sake of others, the Ministry of Health initiated a campaign which called on parents to vaccinate their children in order to protect unvaccinated family members using the slogan "Two drops and the family is protected" to this end. The Ministry of Health chose to provide the public with information about the vaccine without sanctioning parents who decided not to vaccinate their children. In choosing this policy, the Ministry of Health sought to preserve the parents’ right for autonomy. A petition against the vaccination campaign later was submitted to the Supreme Court by an anti-vaccination group. The petitioners claimed that the Ministry of Health was not providing sufficient information about the nature and the dangers of OPV including the fact that the vaccine does not benefit the children who receive it. The Court heard the case on August 29, 2013 and recommended that the petitioners withdraw their petition, which they did [7].

The Global Polio Eradication Initiative’s Independent Monitoring Board noted that "Israel faced a real policy and communications challenge, compounded by the fact that there is a sizable body of anti-vaccination sentiment within the population" [9]. Following a public persuasion campaign, 79% of the children born after 2004 were vaccinated with OPV as well as 69% of children born after 2004 and residing in central of Israel [10]. A 2014 State Comptroller Report stated that the Ministry of Health should draw conclusions from the low compliance rates in certain Israeli regions [11].

**Achieving optimal vaccination uptake rates troubles health policy makers in both Israel and in other countries. The 2013 detection of wild polio in Israeli sewage demonstrates the necessity for interventions aimed at promoting vaccination compliance in cases where persuasion alone has not given rise to an optimal uptake rate.**

**As was mentioned above, the promotion of compliance with OPV had several objectives. However, the following discussion will focus on the legal legitimacy of mandatory vaccination (enforced by criminal sanctions) in the service of the global eradication of polio. This examination is especially important in light of the current public health policy makers’ ambition to eradicate contagious diseases as opposed to past interventions which sought to prevent epidemics.**

**The legal issues raised by the analysis would be relevant to interventions in other cases which seek to attain complete eradication. From a wider perspective, the discussion would be relevant to public health interventions in additional fields, as many of them contain an inherent tension between the ambition of promoting public health and the legal obligation to protect individual rights: "Achieving a just balance between the powers and duties of the state to defend and advance the public health and constitutionally protected rights poses an enduring problem for public health law"** [12].

## Method

A qualitative content analysis research was conducted on relevant court decisions, laws, legislative proceedings, and legislative protocols (all issued or produced between 1948 and 2017). A further analysis was carried out on Health Ministry guidelines and on documented discussions of the Advisory Committee on Infectious Diseases and Immunization.

The study was initiated by analyzing the aforementioned data which was then linked to the relevant theoretical literature such as to attain a cohesive entity. Credibility was established through persistent observation.

## The justification for government intervention in the service of promoting vaccination compliance and the legal means for such interventions

According to L.O. Gostin the public in a democratic society authorizes the government to act for the common welfare. The government thus possesses the sole authority to empower, regulate, or carry out activities designed for the protection or promotion of the general health, safety, and welfare of the population [12]. The IOM emphasizes that "There are solid legal, theoretical, and practical grounds for government in its various forms to assume primary responsibility for the public's health" [12, 13].^1^

The Israel Supreme Court (Justice Barak-Erez) addressed the issue in the 2013 Adalah decision which will be described in detail below [14], and held that the market failure which derives from individual non-vaccination decisions grounded in the notion of “herd immunity” justifies government intervention. Moreover, the Israeli Basic Law: Human Dignity and Liberty (§4) provides that the government has an obligation to protect the life, body, and dignity of every individual. Although the right for health has not been recognized as a basic right, an intervention meant for the eradication of a contagious disease may be considered essential to the protection of human dignity as well as human life and the human body [14, 15].^2^

In attempting to promote vaccination compliance, public health authorities can employ such intervention strategies as client reminders or recalls, the enhancement of access to vaccination services, and the provision of information to target populations or vaccination providers [16, 17]. However, sanctions against individuals who refuse vaccination require specific legislative authorization.

All 50 US states have laws that require vaccination for school admissions. Exemptions vary from state to state, although all school immunization laws grant exemptions to children for medical reasons, and almost all states grant religious exemptions for people who have religious beliefs that prohibit immunizations. 18 states also currently allow philosophical exemptions to those who object to immunization on account of personal, moral or other beliefs [18, 19]; In Canada, three provinces require proof of immunization for school admissions: Ontario, New Brunswick and Manitoba. Exceptions are permitted on medical or religious grounds and for reasons of conscience. Australia’s New Tax System (Family Assistance) Act 1999 states that family tax benefits, child care rebates and child care benefits can only be paid for children who meet immunization requirements. A person may have a medical exemption from vaccination if they are undergoing treatment that compromises their immune system. Religious or conscientious objection is not an exemption category [19–21].

Israel’s Advisory Committee on Infectious Diseases and Immunization (which advises the Israeli Ministry of Health) discussed the possibility of requiring children’s vaccination prior to their admission to the education system in 2008. The committee advised that less intrusive measures should be adopted in order to increase vaccination compliance, and also stated that a mandatory vaccination requirement would not be effective due to enforcement difficulties and the expected number of exemptions that would be granted to parents opposing vaccination. It was therefore decided that a vaccination reminder would be given to all parents who registered their child in an educational institution but that no measures aimed at compelling them to do so would be taken. The possibility of using preschool registration to promote vaccination compliance was re-discussed by the Advisory Committee on Infectious Diseases and Immunization in January 2013. Among other things, the committee discussed the suggestion of requiring a confirmation from a Mother and Child Clinic that the child entering pre-school had been vaccinated in the manner recommended by the Ministry of Health. It also discussed a suggestion requiring parents who oppose vaccination to sign an objection form. Both suggestions were rejected by the committee for several reasons: first, Israeli law does not permit the requirement of vaccinations as a precondition for education; second, the committee believed municipalities would encounter difficulties in implementing the requirement; and third, there was insufficient evidence to indicate that the implementation of such policies would be efficient and that it would promote vaccination compliance [22]. The committee agreed that the Central Vaccination Registry (which did not exist at the time) would be used to remind parents to vaccinate their children and to promote vaccination compliance.

Furthermore, the Israeli Social Security Law of 1995 was amended in 2009 such as to require vaccination in accordance with Ministry of Health recommendations in order to receive an additional child allowance. Ministry of Finance representatives supported the financial sanction and emphasized that it had been proven its effectiveness in other countries. Ministry of Health representatives added that Israel’s unvaccinated population is the reason for disease outbreaks, and that providing parents with a vaccination incentive might promote compliance [23].

A petition against the amendment was later submitted to the Israeli Supreme Court in Adalah Legal Center v. The Israeli Ministry of Social Affairs and Social Services (2013). The petitioners claimed that depriving families with an unvaccinated child of the additional child allowance is a violation of constitutional rights.

In a decision delivered on 4.6.2013 all three judges agreed that the constitutional right to dignity and the constitutional right to autonomy were not being violated in this case. Justice Arbel held that the question of whether the right to autonomy was violated should be answered with respect to the nature of the choice being deprived from the individual and the extent of the coercion applied to this end. The law’s amendment deprives the families of a small financial benefit and does not impose a criminal sanction on parents who refuse to vaccinate their children [24]. Justice Barak-Erez clarified that a financial sanction (unlike a criminal sanction) allows parents the freedom of choosing their actions [25].

As for the constitutional right to equality, Justice Hayut held that legislators are authorized to relate differently to parents who vaccinate their children as opposed to those who refuse to do so [26]. Justice Arbel, on the other hand, was of the opinion that the above distinction is immaterial to the child allowance’s initial purpose – the assurance of minimal financial conditions for survival, meaning that the right to equality is indeed being violated in this case. Nonetheless, Justice Arbel also concluded that this constitutional right violation complies with the stipulations laid down in the limitation clause (§ 8 of the Basic Law: Human Dignity and Liberty) specified hereunder [27].

Justice Barak-Erez did not positively hold that depriving the additional child allowance from families with an unvaccinated child represents a violation of the right to equality, but agreed with Justice Arbel that the law’s amendment complied with the stipulations provided in the Limitation Clause: The amendment has a proper purpose (to protect unvaccinated children and promote public health); there is high probability that a financial sanction would be effective and promote vaccination compliance; and the intervention is both minimally infringing and proportionate since it has been balanced by the parents’ right to opposition and appeal [28].

However, the additional child allowance was later cancelled, and the amendment to the Israeli Social Security Law was repealed by the Israeli parliament before its implementation [29].

The Public Health Ordinance enacted in 1940, is currently the only reference in Israeli law to public health interventions. According to §19 of the Ordinance (which was translated from Palestine Gazette Extraordinary No. 1065 of 20th December, 1940 - Supplement No. 1) "In any town, village or area where an infectious disease assumes or is likely to assume an epidemic character or where there exists in the neighborhood infectious disease such as in the opinion of the Director constitutes a danger to the public health of such town, village, or area, the Director or Medical Officer may proceed to take such measures to protect the inhabitants thereof from infection as he considers necessary and may for this purpose inter alia subject the inhabitants of such town, village or area to such prophylactic inoculation or vaccination as in his opinion is necessary to limit the spread of infection. Any person who willfully refuses to submit to inoculation or vaccination under this section…is guilty of an offence and is liable to a fine not exceeding five pounds or imprisonment for a term not exceeding one month." §20 of the Ordinance is an emergency powers provision which relates to a formidable epidemic, or to an endemic or infectious disease which threatens “any part of Palestine” and empowers the High commissioner to order "any such matters or things as may appear advisable for preventing or mitigating such disease", including "the prophylactic inoculation or vaccination of the general public" [30]. Such mandatory vaccination as provided by the Ordinance was only imposed twice in Israeli history: once in 1949, when Israel faced a smallpox outbreak, and once in 1994 when a measles outbreak occurred (mainly in the Negev region) [31].

In light of the above, government intervention in the service of promoting vaccination compliance is thus theoretically justified. However, current Israeli law does not follow other jurisdictions with respect to the imposition of sanctions on those who refuse routine vaccination but rather only allows the imposition of sanctions in the specific circumstances provided by the 1940 Ordinance.

## Was it legally legitimate to impose OPVs in 2013 in accordance with the public health ordinance, 1940?

As mentioned above, the detection of wild poliovirus in Israeli sewage led the Ministry of Health to initiate a massive public health campaign aimed at persuading parents to vaccinate their children with OPV. Considering the State Comptroller’s disapproval of the low compliance rates in certain Israeli regions, could the Ministry of Health have legally considered more intrusive measures of imposing OPVs in accordance with the Ordinance?

The term “epidemic,” which justifies the implementation of the Public Health Ordinance and the imposition of mandatory vaccination, refers to "the occurrence in a community or region of cases of an illness, specified health behavior, or other health related events clearly in excess of normal expectancy" [32]. Given that the majority of the Israeli population was previously immunized against polio with either OPV or IPV, and since no morbidity incidences had occurred since 1988, it may be argued that even one case of morbidity would be “in excess of normal expectancy.”

The question of whether the detection of wild poliovirus in Israeli sewage was also a threat to public health is difficult to answer, as the Ordinance does not specify the severity of the risk to public health required for its implementation. According to L.O. Gostin, only a “significant” risk should be perceived as a threat to public health, as opposed to a speculative, theoretical or remote risk [12]. The risk of polio contamination in Israel in 2013 could have been perceived as significant, as polio viruses are highly contagious and spread by the fecal–oral route. Although the probability of harm as a result of a polio infection is low, the severity of harm that an unvaccinated individual or an individual with a suppressed immune system may suffer (permanent paralysis) is high.

Nonetheless, it seems that both §19 and §20 of the Ordinance authorize the health authorities to impose mandatory vaccination when there is a significant risk to the **local population** and thus they do not relate to legitimate interventions required for the global eradication of a disease. Almost all individuals in Israel were protected from the clinical polio disease in 2013 [10], and there was no risk to the local population (as opposed to the risk of a single case of morbidity). Hence, both sections of the Ordinance could not provide a legal basis for compulsory OPV.

## Would it be legally legitimate to impose OPVs in accordance with a new public health law?

The Israeli Association of Public Health Physicians along with the Israeli Medical Association recently made efforts toward the legislation of a new Public Health Law which would replace the antiquated sections of the 1940 Ordinance (in a manner akin to public health law reforms in other countries as "existing statutes are outdated, contain multiple layers of regulation, and are inconsistent" [33]). Moreover, the minority opinion in the Adalah case held that the entire domain of vaccination should be addressed by new legislation [26].

It is therefore essential to examine the legitimacy of legislation that would authorize the health officials to not only impose mandatory vaccination where there is a significant risk to the local population (or the risk of an epidemic) but also where the intervention seeks to promote the eradication of a disease. The present examination relates to an obligation which, like §19 of the Ordinance, would be enforced by the criminal sanctions of a financial penalty or imprisonment for no longer than a month.

Any authorization granted to health officials under a new Public Health Law must comply with the provisions of the 1992 Basic Law: Human Dignity and Liberty. This Basic Law states that no violation of the life, body or dignity of any person should occur except in accordance with the Limitation Clause, which will be discussed later.

The constitutional right to dignity includes, according to Israeli Supreme Court judgements, the right to autonomy [34]. One aspect of the right to autonomy is parental autonomy, which refers to parents’ right and obligation to take care of their minor children. The rational for parental autonomy is the natural bond between parents and children, and the underlying presumption is that parents will generally make the best decisions for their children. Moreover, it is appropriate to let parents decide when they are the ones who will bear the consequences of their decisions [14, 35].

### Do mandatory OPVs enforced by criminal sanctions violate the right to parental autonomy?

The right to autonomy in the medical context is implemented through the requirement of “informed consent” prior to medical interventions. The “informed consent” doctrine consists of two components: The physician’s duty to disclose information about the prospects and risks of the procedure (informed participant) and the patient’s right to freely consent or refuse to the treatment (informed choice) [36, 37].

It may be argued that this liberal interpretation of bioethics which regulates curative medicine and proceeds from an assumption of absolute bodily autonomy does not apply to public health interventions. Childress et al. stated that "It would be a mistake to suppose that respect for autonomy requires consent in all contexts of public health" [38]. While curative medicine deals with the health of an individual, public health interventions deal with the health of a population. A population’s interests may sometimes contradict individual interests and justify interventions which do not assure an individual’s consent or despite her or his refusal [36–38]. Moreover, it is unrealistic to obtain informed consent to a public health intervention when the health professional cannot predict whether a specific unvaccinated individual will benefit from the intervention in the future. This is because those members of the population who would stand to gain from the intervention are unknown, and their number can only be estimated in advance [39]. The legitimacy for exercising state power without receiving “informed consent” derives from Social Contract Theory, which suggests that people agree to accept certain obligations by choosing to live in a society. The presumption of obligation acceptance is based on the “tacit consent” of an individual who resides in the state to government rule in exchange for the benefits of society. Other sources for the presumption of obligation acceptance are the “hypothetical consent” of an individual to be bound by the state which is necessary for social functioning, as well as a fairness of balancing the state’s benefits to the individual against the limits that are necessary for maintaining those benefits [36, 37, 40].

The Israeli Supreme Court decision in the case of Juhar Aturi v. The Israeli Ministry of Health (1993) related to the duty to disclose vaccine risks, and held that informed consent for vaccination does not require the disclosure of remote and rare side effects. Latter Israeli court decisions expanded the duty of disclosure in curative medicine but did not relate to preventive medicine and vaccinations. A limited disclosure requirement might lead to a limited requirement for individual consent to vaccination (or a limited implementation of the informed consent doctrine in public health interventions) as any discussion on the duty of disclosure cannot be separated from a discussion on the right to free consent [41].

Nonetheless, the Israeli Patient’s Rights Law of 1996 espoused the “informed consent” doctrine with respect to both the curative medical context as well as preventive treatment. According to the law, medical treatment, which includes preventive treatment, shall not be given to a patient without her or his “informed consent.” A 2005 decision by the Israeli District Court related specifically to vaccination and clearly stated that the “informed consent” requirement applied to a decision on vaccination just as it applied to a decision on any other medical procedure [42].

The Supreme Court’s decision in the Adalah case addressed the circumstances in which the parental autonomy to determine whether or not their children should be vaccinated was violated. The court related to obligatory vaccination enforced through criminal sanctions (whose legitimacy is examined here in the OPV context) as hard paternalism (unlike the deduction of an additional child allowance which is soft paternalism). As such, the court held that it violated the right to parental autonomy [14].

### Can the violation of parental autonomy be justified in the 2013 circumstances?

According to John Stuart Mill, the right to autonomy or parental autonomy (although applicable in public health interventions) is not unlimited: persons should be free to think, speak and behave as they wish, provided they do not interfere with a like expression of freedom by others (“the harm principle”) [43]. L.O. Gostin interprets this as suggesting that personal freedoms extend only so far as they do not intrude on the health, safety and other legitimate interests of other individuals. Persons, according to Gostin, are entitled to live without the risk of serious injury or disease [12, 44].

The famous U.S. Court decision in Jacobson v. Massachusetts (1905) followed the Millian doctrine and justified a law that mandated vaccination despite restricting liberty: In 1809, Massachusetts was the first state in the U.S. to compel vaccination against smallpox. According to the state law, any refusal to the smallpox vaccination resulted in penalties ranging from fines to imprisonment. Henning Jacobson refused both the vaccination and the payment of a $5 fine. Jacobson argued before the U.S. Supreme Court that the Massachusetts law violated the due process and equal protection provisions of the Fourteenth Amendment ("nor shall any state deprive any person of life, liberty or property without due process of law.") Jacobson further alleged that it was unreasonable for the state to interfere with his liberty when he had not been taken with any illness. The US Supreme Court decided in favor of Massachusetts in 1905, declaring that the state had the authority to enact health laws of every description to guard the common good in whatever way the citizens, through their elective representatives, thought appropriate: "Even liberty itself, the greatest of all rights, is not [an] unrestricted license to act according to one's own will" [45].

The violation of parental autonomy can be justified according to Israeli law if it complies with the stipulations mentioned in the Limitation Clause (§8 of the Basic Law: Human Dignity and Liberty): the infringement is carried out according to a law befitting the values of the State of Israel, is enacted for a proper purpose and to an extent no greater than is required.

#### Is a law which authorizes health officials to mandate OPVs in order to eradicate polio enacted for a proper purpose?

Health economists have justified interventions aimed toward increased vaccination coverage through cost-benefit, cost-effectiveness and cost-utility analyses – techniques for quantifying and measuring the value of an intervention by weighing the likely costs, including the consequences of adverse events, against the potential positive outcomes. Given that the eradication of contagious diseases reduces medical care expenses and adds years of productive life to members of society for a small per-person cost, an increased compliance with OPVs with a view to the eradication of polio is considered a proper purpose according to the aforementioned economic-theoretical methods [46, 47].^3^

However, economic methods, which help in the determination of public health policy, and especially in cases of limited public health resources, do not reflect moral considerations and preferences that may also justify the violation of individual autonomy. One of these moral considerations is social justice, which is a commitment to the attainment of a sufficient level of health for all [48, 49]. The eradication of polio such as would protect the unimmunized population correlates with these social justice values. Moreover, the Syrian Civil War that was still raging in 2013 made it increasingly more difficult for Syrians to access medical services and vaccines. Many Syrian residents and refugees were not vaccinated against polio and were at risk of poliovirus infection. The promotion of polio eradication in the region under these circumstances would have the potential of protecting the vulnerable Syrian population. Protecting population health (as opposed to community health) without national or geographical limitations correlates with the “global justice” which is required in a globalized world where communicable diseases can easily cross borders [50].

The promotion of polio eradication in Israel may also be considered a proper purpose that would justify the infringement of individual autonomy given that the GPEI – the Global Polio Eradication Initiative spearheaded by national governments, the WHO, Rotary International, the U.S. CDC and UNICEF supported by the Bill and Melinda Gates Foundation have striven toward the eradication of the disease since 1988 [51].^4^ Israel is thus morally and politically obliged to participate in the global effort toward the eradication of polio. Another case in which a disease was declared as a global health threat by the WHO was when Severe Acute Respiratory Syndrome (SARS) was diagnosed in 8098 people in 26 countries and caused 774 deaths. China, in which the disease had first been diagnosed, was criticized by the WHO and by other countries for delays in reporting cases and for a lack of cooperation with the WHO [52, 53]. Israel would thus not be able to risk its position as a developed country which cooperates with the global effort toward the eradication of polio.

#### Does a mandatory OPV enforced by criminal sanctions violate autonomy to an extent no greater than is required?

The term “no greater than required,” which justifies an intervention despite potentially violating the right to autonomy relates to 3 sub-terms: the effectiveness of the intervention (rational connection); the least infringing intervention, and the proportionality between the benefits from the intervention and the concomitant infringement of human rights.

##### Would a mandatory OPV be an effective intervention and promote the eradication of polio?

In order to determine whether a mandatory OPV enforced by criminal sanctions would be an effective intervention, it is necessary to clarify when an intervention meant for the promotion of OPV compliance would be considered “effective”.

As was mentioned above, the Ministry of Health advised all Israeli parents to vaccinate children who were born after 2004 with OPV in 2013. The public health campaign which followed this recommendation sought to attain maximum compliance. However, the State Comptroller criticized the Ministry of Health for low compliance rates since 79% of the children born after 2004 were vaccinated as well as only 69% of those children born after 2004 and residing in Central Israel. This begs the question of whether an intrusive intervention would result in higher compliance rates. In this respect, it should be noted that Israeli case law suggests that there is no need to prove that the intervention would surely attain its objective, and that it suffices to prove reasonable probability [54].

The effectiveness of compulsory OPVs in Israel depends by and large on the reasons for low compliance. Low compliance rates which derive from the vaccination hesitancy of Israeli parents who seek an open and trusting relationship with their health care providers and who wish to make autonomous decisions regarding vaccination would not be increased by sanctions [55, 56]. Sanctions would also be certain to provoke Israeli parents who already believe that the government is too intrusive with respect to their freedoms as well as parents who are convinced that the vaccine would endanger their child^5^.^6^

Beside concerns that sanctions would not stimulate hesitant parents as well as parents who oppose government interference, the sanctions’ effectiveness would likely be reduced by enforcement difficulties: imposing the obligation to follow, register and report the immunization status of every Israeli child would require additional budgetary allocations to the health system. The lack of such an additional budget would interfere with the attainment of the stated objective.^7^

Over and above budgetary shortfalls, the imposition of sanctions on parents who refuse to vaccinate their children also involves legal and ethical issues associated with the registration of unvaccinated children. The Israeli Privacy Protection Law (1981) forbids the disclosure of an individual’s private matters (including medical information), although a violation of this privacy is permitted when it is done in accordance with a valid legal provision. The Public Health Ordinance (in §65b) authorizes the Minister of Health to establish a national immunization registry and thus legitimizes the disclosure of vaccination statuses [57]. However, the ethical dilemma that exists between the violation of healthy people’s medical confidentiality and the promotion public health remains and requires an in depth discussion in and of itself. Moreover, the registry’s legal objectives were the supervision of vaccines administered at public Mother and Child Clinics, HMOs (Health Maintenance Organizations) and schools as well as the implementation of §68 of the National Insurance Law, which deprived an additional child allowance from the non-vaccinated. The implementation of child allowance reductions is no longer relevant as the associated legal amendment has been repealed. The registry’s sole objective at present is thus the supervision of the vaccines administered to the population. Using these records in order to impose sanctions on unvaccinated children would deviate from this objective and would very likely provoke opposition [58].

##### Is a mandatory OPV enforced by criminal sanctions the least autonomy- infringing intervention?

If we were to overcome parental opposition and enforcement difficulties, and conclude that a mandatory OPV would be an effective intervention for promoting compliance and eradicating polio, we must examine whether the enforcement of OPVs through criminal sanctions would also be the least autonomy-infringing intervention from an effectiveness perspective. According to Childress et al. [38], "The fact that a policy will infringe a general moral consideration provides a strong moral reason to seek an alternative strategy that is less morally troubling".^8^

Reviews of evidence regarding interventions which sought to improve vaccination coverage in children, adolescents and adults, hold that strong scientific evidence supports the assumption that non-intrusive interventions (i.e. client or provider reminder/recall or expanded access to health care settings) can be effective enough in improving vaccination coverage [16]. In the Adalah case [14], both Justice Arbel and Justice Barak-Erez held that deducting an additional child allowance from parents who refuse to vaccinate their children is the least infringing intervention that would promote vaccination compliance, and that a criminal sanction would surely be more intrusive.

However, the aforementioned reviews of evidence and the Adalah decision relate to routine vaccinations which aim to protect the individual and ensure herd immunity, and do not relate to vaccines recommended for disease eradication where there is no risk of a local outbreak. Expecting parents to expose their children to vaccination in order to eradicate a disease worldwide has low prospects given the extent of the expected opposition for an intervention with such remote outcomes.

It may therefore be argued that a mandatory OPV backed by criminal sanctions would be the least autonomy-infringing intervention necessary for attaining a high degree of compliance.^9^

Nonetheless, the health authorities must conclude that educating the public regarding all aspects of the importance of polio eradication, including the negative political outcomes of a refusal to participate in the global polio eradication initiative, is ineffective before implementing sanctions (let alone criminal sanctions) against parents who refuse to vaccinate their children with OPV. The obligation to use non-intrusive measures before enforcing vaccinations through sanctions accords with the concept of Therapeutic Jurisprudence (TJ), which suggests that legislation should be the last resort after the public has been provided with relevant information such as to build trust and promote compliance [31].^10^

##### The requirement of proportionality

The discussion above relates to a new Public Health Law that would authorize health officials to enforce a mandatory vaccination in the service of promoting disease eradication and to enforce this obligation throughout a financial penalty or imprisonment of no longer than a month.

A financial penalty (unlike the deprivation of freedom) might be considered as a tool for prompting action.^11^ However, the proportionality of a decision to convict an individual who refuses vaccination requires a clearing of this individual’s criminal record once she or he complies with the obligation to vaccinate. Moreover, this provision must also include a procedure which would discuss requests for exemptions. In this respect the granting of exemptions in cases of medical contraindications alone would not diminish the Law’s proportionality (as the granting of religious or philosophical exemptions might render the law ineffective).

Nonetheless, a decision to enforce a mandatory OPV by a financial penalty in the service of globally eradicating polio even if the aforementioned stipulations are met, might be perceived as incompatible with the violation of the parental autonomy to refuse to altruistically vaccinate a healthy child who is not at risk for a clinical disease (Not only was there no risk of a polio outbreak in Israel in 2013, but the OPV vaccination recommendation was also given to children who had already been vaccinated with IPV and had possessed humoral protection against polio).

Enforcing the vaccination through a “softer” sanction (i.e. the deprivation of some child benefits – as was suggested in 2009 in order to promote compliance with routine vaccination in Israel), might not attain a maximum degree effectiveness but would provide parents with the genuine discretion of deciding whether or not to participate in the global eradication efforts, and may thus be considered as proportionate to the concomitant violation of parental autonomy [14].

## Conclusions and recommendations for a new public health law

The global ambition of eradicating contagious diseases and totally preventing morbidity requires health authority interventions in order to promote vaccination compliance.

An examination of the legal legitimacy for a mandatory OPV accompanied by criminal sanctions in the service of polio eradication reveals that such intervention would infringe autonomy to an extent greater than required: although eradication is a proper purpose, criminal sanctions might not be effective and may even provoke resistance. Moreover, and even if we were to overcome parental opposition and enforcement difficulties, criminal sanctions would still not be the least-infringing intervention when a public education campaign would achieve the intervention’s objectives, and would not be proportionate when the recommended vaccine has remote benefits.

The appropriate intervention for promoting vaccination compliance in the service of eradicating contagious diseases should start with nonrestrictive measures such as enhancing the accessibility of vaccination, providing the public with complete and relevant information about the vaccine, or offering incentives to parents who comply with vaccination recommendations.

However, in situations where nonrestrictive measures would not suffice in the attainment of health authority objectives, there may be some added value inherent in a law which would authorize it to enforce a mandatory vaccination.

Such a Law should also include several sanctions meant for the enforcement of obligatory vaccination, i.e., tiers of financial sanctions, and the establishment of a criminal record or the quarantine of individuals who refuse vaccination. According to the “intervention ladder” theory [59], these sanctions should be formulated from least restrictive to most restrictive.^12^ Such a formulation would in turn require the evaluation of the extent of intrusiveness inherent in every such sanction by experts in law and ethics.

The suggested Law should further describe the circumstances which justify the implementation of every sanction: a disease in close proximity represents a risk to public health as a significant part of the population is unimmunized; it is necessary for the promotion of compliance with routine vaccination; or the WHO recommends that the population be vaccinated in the service of promoting global objectives. Health authorities should also be granted the discretion to decide on the least restrictive sanction in unexpected circumstances.

The terms employed by legislators must also be interpreted. In this respect, and if the law only justifies criminal sanctions when the virus represents a risk to the population, then the term “risk” requires the clarification of its severity and nature,^13^ and the term “population” requires the clarification of its geographic borders.

The core of the new law should also contain a description of the decision-making process which must be based on facts and which must assure fairness to the individual whose rights are being infringed^14^ [60].

Finally, the public should be entitled to participate in the decision-making process or at least be allowed to follow its fully transparent proceedings, since the acquisition of public justification would diminish public resistance to the intervention and consequently increase its effectiveness [38].

## Abbreviations

CDC: US Centers for Disease Control and Prevention
IOM: Institute of Medicine
IPV: Inactivated Polio Vaccine
MMRV: Measles, Mumps, Rubella, Varicella
NII: National Insurance Institute of Israel
OPV: Oral Polio Vaccine
WHO: World Health Organization

## Footnotes

1 L.O. Gostin stresses that a political or governmental entity possesses principle responsibility to protect and promote public health, as “public health can be achieved only through collective action, not individual endeavor. Acting alone, individuals cannot ensure even minimum levels of health. Individuals may procure personal medical services…yet no single individual or group of individuals can ensure the health of the community” [61].

2 Any government intervention meant for the protection of health must comply with the stipulations mentioned in the Limitation Clause (§8) if other basic rights are being violated such as the right to individual autonomy as discussed in the present article.

3 Economic theories assume rational behavior among vaccine recipients, even though this assumption is not always true (some people may prefer to pay a fine rather than vaccinate their children). Therefore, and although these theories may consider the eradication of contagious diseases as a proper purpose, they cannot always support the effectiveness of the intervention implemented with a view to accomplishing this purpose, as discussed in the present article.

4 The WHO and UNICEF declared a public health emergency in response to the detection of wild poliovirus in environmental samples taken in Egypt, Israel, the West Bank and Gaza Strip following a polio outbreak in Syria. It was noted that a multi-country response was needed despite the fact that polio cases had only been detected in Syria, given the ongoing civil war in this country and the mass displacement of its population into neighboring countries. “The primary goal is to ensure that oral polio vaccine (OPV) is urgently delivered into all communities” [51].

5 Sanctions applied against those who refuse vaccination (depriving the right to education) in the US resulted in increased immunization rates [16, 62, 63]. However, given that the majority of the population in Israel complies with vaccination recommendations voluntarily, and in light of Israeli parents’ motivation toward making autonomous decisions, the imposition of a mandatory vaccination may result in resistance and attain the opposite of its intended purpose [56, 64].

6 The enactment of the Vaccination Act in England (1853), which imposed fines on parents who failed to allow their children to be vaccinated, led to riots in the streets and to serious protests made not only by those opposing the vaccination itself but also by opponents of government intrusion on personal autonomy [19, 20].

7 The work of ensuring that all US students were vaccinated according to school admission laws required the cooperation of both health and education administrators with different priorities. School principals had difficulty keeping track of students’ medical records and claimed that budget shortages prevented the implementation of enforcement measures [19].

8 Gostin suggests that adoption of equally effective and less restrictive alternatives would also encourage voluntary compliance [60].

9 Imposing tort liability on parents who refuse to vaccinate their children may also encourage vaccination. However, such liability may only be imposed when the parents’ choice of non- vaccination results in harm to others. Proving that a particular unvaccinated child transmitted a disease to another and caused harm can be a difficult and in some cases an even impossible task [65].

10 In the Adalah case, Justice Barak-Erez (in ¶65 of the Court’s decision) held that public education was essential to promoting compliance with vaccination, and referred to Alberstein M, Davidovitch N. .Therapeutic Jurisprudence and Public Health: Israeli Perspectives. Bar Ilan studies. 2010; 26: 549, which called for the implementation of therapeutic Jurisprudence in public health [31].

11 The minority opinion in the Adalah case considered the partial deprivation of child benefits as a financial sanction which would be legitimate as part of a general piece of legislation that would address vaccination issues [26].

12 According to the Nuffield Council of Bioethics, the “intervention ladder” relates to public health interventions in general, and includes both intrusive and nonintrusive interventions which do not require legislation.

13 The law should clarify the risk which justifies a certain sanction according to the mode of transmission, the risk’s duration, the probability of harm and the severity of harm [12].

14 The Israeli Mental Healthcare Law 1991, which replaced a former law passed in 1955, is an example of a statute that balances society’s interest in protecting the individual or the public from the symptoms of mental illnesses against the need to promote human rights and individual autonomy. Among other things, the law provides for limited psychiatric discretion in the imposition of forced hospitalization, and further provides for the option of appealing a psychiatric decision, as well as the entitlement to legal counseling (for the individual facing hospitalization).
